# Ternary Eutectic Ezetimibe–Simvastatin–Fenofibrate System and the Physical Stability of Its Amorphous Form

**DOI:** 10.1021/acs.molpharmaceut.1c00485

**Published:** 2021-08-22

**Authors:** Justyna Knapik-Kowalczuk, Daniel Kramarczyk, Karolina Jurkiewicz, Krzysztof Chmiel, Marian Paluch

**Affiliations:** † Faculty of Science and Technology, Institute of Physics, University of Silesia in Katowice, SMCEBI, 75 Pułku Piechoty 1a, 41-500 Chorzów, Poland; ‡ Department of Pharmacognosy and Phytochemistry, School of Pharmacy with the Division of Laboratory Medicine in Sosnowiec, Medical University of Silesia in Katowice, Jagiellonska 4, 41-200 Sosnowiec, Poland

**Keywords:** amorphous pharmaceuticals, physical stability, ternary eutectic, eutectic system, ezetimibe, simvastatin, fenofibrate

## Abstract

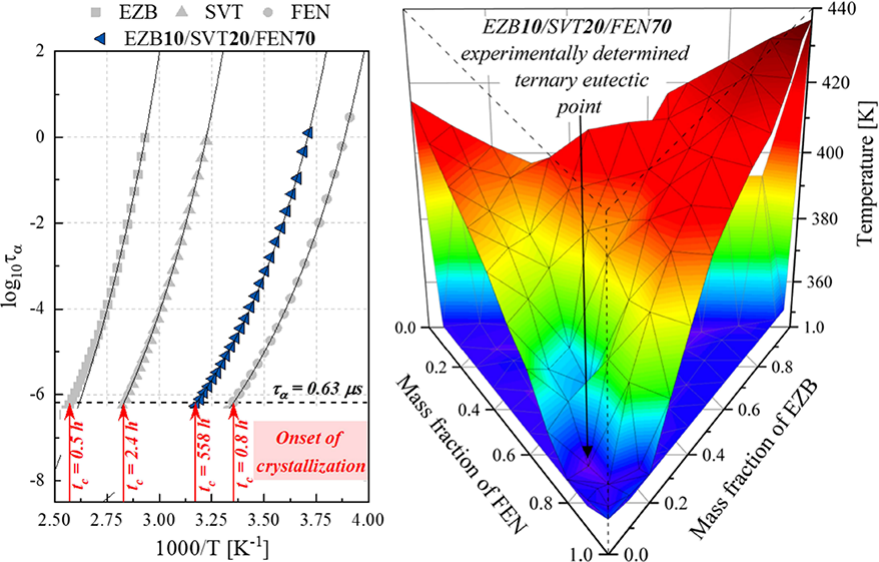

In this study, the phase diagram of the ternary system of ezetimibe–simvastatin–fenofibrate was established. It has been proven that the ternary composition recommended for the treatment of mixed hyperlipidemia forms a eutectic system. Since eutectic mixtures are characterized by greater solubility and dissolution rate, the obtained result can explain the marvelous medical effectiveness of combined therapy. Considering that another well-known method for improving the aqueous solubility is amorphization, the ternary system with eutectic concentration was converted into an amorphous form. Thermal properties, molecular dynamics, and physical stability of the obtained amorphous system were thoroughly investigated through various experimental techniques compared to both: neat amorphous active pharmaceutical ingredients (considered separately) and other representative concentrations of ternary mixture. The obtained results open up a new way of selecting the therapeutic concentrations for combined therapies, a path that considers one additional variable: eutecticity.

## 1 Introduction

Mixed hyperlipidemia is a significant risk factor for the development of cardiovascular diseases.^1^ Dietary modification and increased physical activity are the first recommended therapy to modify the abnormal level of the lipids in the bloodstream.^2^ Unfortunately, in many cases, lifestyle changes are not enough to overcome this problem. Therefore, besides a healthy diet, regular physical activity, alcohol abstinence, and smoking cessation, the use of lipid-lowering drugs is needed.^3^

There are many active pharmaceutical ingredients (APIs) reducing the level of serum triglycerides and cholesterol. Among them, one can distinguish (i) statins, which inhibit 3-hydroxy-3-methylglutaryl coenzyme A reductase, and thereby block the cholesterol synthesis;^4,5^ (ii) fibrates, which activate peroxisome proliferator-activated receptors leading to induce the transcription of genes facilitating lipid metabolism;^6^ and (iii) cholesterol absorption inhibitors working in the intestinal brush border membrane such as ezetimibe (EZB).^7^ Despite a large number of cholesterol-lowering APIs, the National Cholesterol Education Program Adult Treatment Panel III highlights that the monotherapy of any known drug might not be sufficient to treat mixed hyperlipidemia properly.^8^ Consequently, combined drug therapy is recommended.^9^

It has been proven, that coadministration of fenofibrate (FEN) and statin is more effective in controlling atherogenic dyslipidemia in patients with type 2 diabetes mellitus, metabolic syndrome, or mixed dyslipidemia than the administration of either drug alone.^10,11^ In a similar patient population, the lipid-lowering effects on low-density lipoprotein cholesterol (LDL-C) and triglycerides were significantly greater with the coadministration of EZB and FEN compared to each monotherapy.^12^ Moreover, a multitude of clinical studies proved that the addition of a cholesterol absorption inhibitor to statin therapy reduces LDL-C and total cholesterol levels much more effectively than at least a double dose of statin.^13^ As Bays et al. showed that the coadministration of 10 mg of EZB and 10 mg of simvastatin (SVT) results in a similar reduction in plasma LDL-C level to 80 mg of SVT alone.^14^ Since any binary combination of statins, fibrates, and cholesterol absorption inhibitors brings improved medical effectiveness in comparison to the monotherapy, Farnier et al. suggested treating mixed hyperlipidemia with a ternary drug composition.^2^ Clinical trials on 611 patients have shown that coadministration of SVT (20 mg), EZB (10 mg), and FEN (160 mg) effectively improved the overall atherogenic lipid profile, which could not be achieved in mono or binary therapy.

Both EZB, SVT, and FEN belong to Biopharmaceutics Classification System (BCS) class II.^15−19^ It means that all these APIs, on the one hand are characterized by good permeability, but on the other hand, have a problem with poor aqueous solubility. Low water solubility and slow dissolution rate of crystalline APIs in the gastrointestinal tract are the major obstacles impeding the development of drug formulations for oral delivery.^20,21^ Therefore, many efforts are currently devoted to improving the solubility-limited bioavailability of marketed BCS class II pharmaceuticals.^22−24^ Several methods might help in reaching this goal.^25^ One of them is based on the formation of eutectic systems.^26^ A eutectic mixture is defined as a mixture of two or more components that do not interact to form a new chemical compound but, at certain ratios are miscible in the molten state, usually at a temperature lower than the melting points of either of their constituents.^27^ It has been many times reported that eutectic concentrations play a key role in improving the absorption of many APIs by increasing their solubility and dissolution properties.^28,29^ With that in mind, one might wonder whether or not the marvelous therapeutic properties observed by Farner et al. during the combined therapy with EZB, SVT, and FEN are connected with the accidental employment of the ternary eutectic concentration of these APIs. Finding the answer to this question is the first goal of this paper. To achieve this aim, the ternary phase diagram of EZB, SVT, and FEN was determined both experimentally and theoretically.

It has to be mentioned that another very well-known path leading to the water solubility improvement of the crystalline APIs is their conversion to amorphous form.^30−37^ Thus, the second goal of this paper is to prepare and characterize the disordered counterparts of the investigated ternary compositions. Working with amorphous APIs, one cannot forget that such systems have unfortunately, one disadvantage blocking their widespread use. Namely, such systems are physically unstable, and thus during the time of manufacturing, transportation, or storage, they might revert to their initial, i.e., crystalline form.^38−42^ Hence the third goal of this study was to examine the effect of eutectic concentration on the physical stability improvement of the ternary amorphous system. To achieve this goal, a series of: (i) short-term time-dependent dielectric experiments at elevated temperature conditions as well as (ii) long-term diffraction studies under standard storage conditions were performed.

## 2 Materials and Methods

### 2.1 Materials

**Figure 1:**
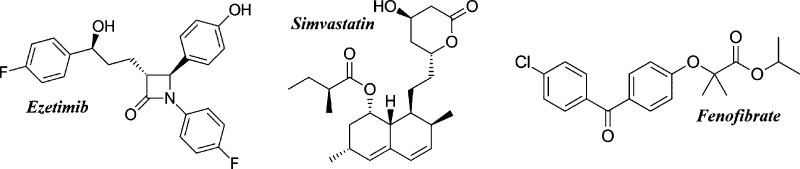
Chemical structures of EZB, SVT, and FEN.

EZB of purity greater than 99% and molecular mass *M*_w_ = 409.4 g/mol was purchased from Polpharma (Starogard Gdański, Poland). This pharmaceutical is described chemically as ((3*R*,4*S*)-1-(4-fluorophenyl)-3-[(3*S*)-3-(4-fluorophenyl)-3-hydroxypropyl]-4-(4-hydroxyphenyl) azetid in-2-one). SVT of purity greater than 98% and molecular mass *M*_w_ = 418.6 g/mol was purchase from ATOMAX and is described chemically as butanoic acid, 2,2-dimethyl-(1S,3*R*,7*S*,8*S*,8a*R*)-1,2,3,7,8,8a-hexahydro-3,7-dimethyl-8-[2[(2*R*,4*R*)tetra hydro-4-hydroxy-6-oxo-2*H*-pyran-2-yl] ethyl]-1-na-phthalenyl ester. FEN, which is described chemically as 2-[4-(4-Chlorobenzoyl) phenoxy]-2-methylpropanoic acid isopropyl ester (*M*_w_ = 360.83 g/mol), was purchased from Sigma-Aldrich with purity greater than 99%. All neat APIs were used as received. Their chemical structures are presented in Figure 1.

### 2.2 Differential Scanning Calorimetry

The thermal properties of neat, both crystalline and amorphous EZB, SVT, and FEN drugs, as well as their binary and ternary systems were examined by a Mettler–Toledo DSC 1 STAR^e^ System (Columbus, OH, USA) equipped with an HSS8 ceramic sensor and 120 thermocouples. The instrument was calibrated for temperature and enthalpy using indium and zinc standards. The melting point was determined as: (i) the onset temperature in the case of neat APIs and the solidus transition in the mixtures, or (ii) the peak maximum temperature in the case of the mixture’s liquidus transition. At the same time, the glass transition temperature (*T*_g_) was determined as the midpoint of the heat capacity increment. The samples were measured in an aluminum crucible (40 μL). All crystalline mixtures were measured with a rate of 10 K/min, while during the studies of amorphous samples, two heating rates (5 and 10 K/min) were employed. Each experiment was at least performed in duplicate.

### 2.3 X-ray Diffraction

Long-term X-ray diffraction (XRD) experiments were performed for ternary amorphous samples stored at room temperature and relative humidity RH ≈ 40% on a Rigaku-Denki D/MAX RAPID II-R diffractometer (Rigaku Corporation, Tokyo, Japan) equipped with a rotating Ag anode, an incident beam (002) graphite monochromator, and an image plate detector in the Debye–Scherrer geometry. The wavelength of the incident beam λ was 0.5608 Å. The pixel size was 100 μm × 100 μm, and the beam width on the sample was 0.3 mm. Measurements were performed for powder samples enclosed in borosilicate glass capillaries, and the background intensity from empty capillary was collected and subtracted. The two-dimensional diffraction patterns were converted into one-dimensional intensity data versus the scattering angle. The temperature-dependent XRD measurements were performed using an Oxford Cryosystem 800 unit.

### 2.4 Broadband Dielectric Spectroscopy

The molecular dynamics and the time-dependent isochronal physical stability of the selected ternary amorphous EZB/SVT/FEN systems were measured utilizing a Novocontrol GMBH Alpha dielectric spectrometer (Montabaur, Germany). Dielectric spectra were registered in a broad frequency range from 10^–1^ to 10^7^ Hz. During the nonisothermal dielectric experiments, the samples were heated from temperature *T = T*_g_ to *T = T*(τ_α_*=* 0.2 ns) with a step of 2 K. While during isothermal (time-dependent) studies, the samples were stored under defined *T* conditions (specific for each sample) for a longer period of time during which spectra were registered every 600 s. The temperature was controlled by a Quattro controller with temperature stability better than 0.1 K. The systems were measured in a parallel-plate cell made of stainless steel (diameter of 15 mm, and 0.1 mm gap provided by silica spacer fibers).

## 3 Results and Discussion

### 3.1 Determination of Binary Eutectic Mixtures of EZB-SVT, EZB-FEN, and SVT-FEN Systems

**Figure 2:**
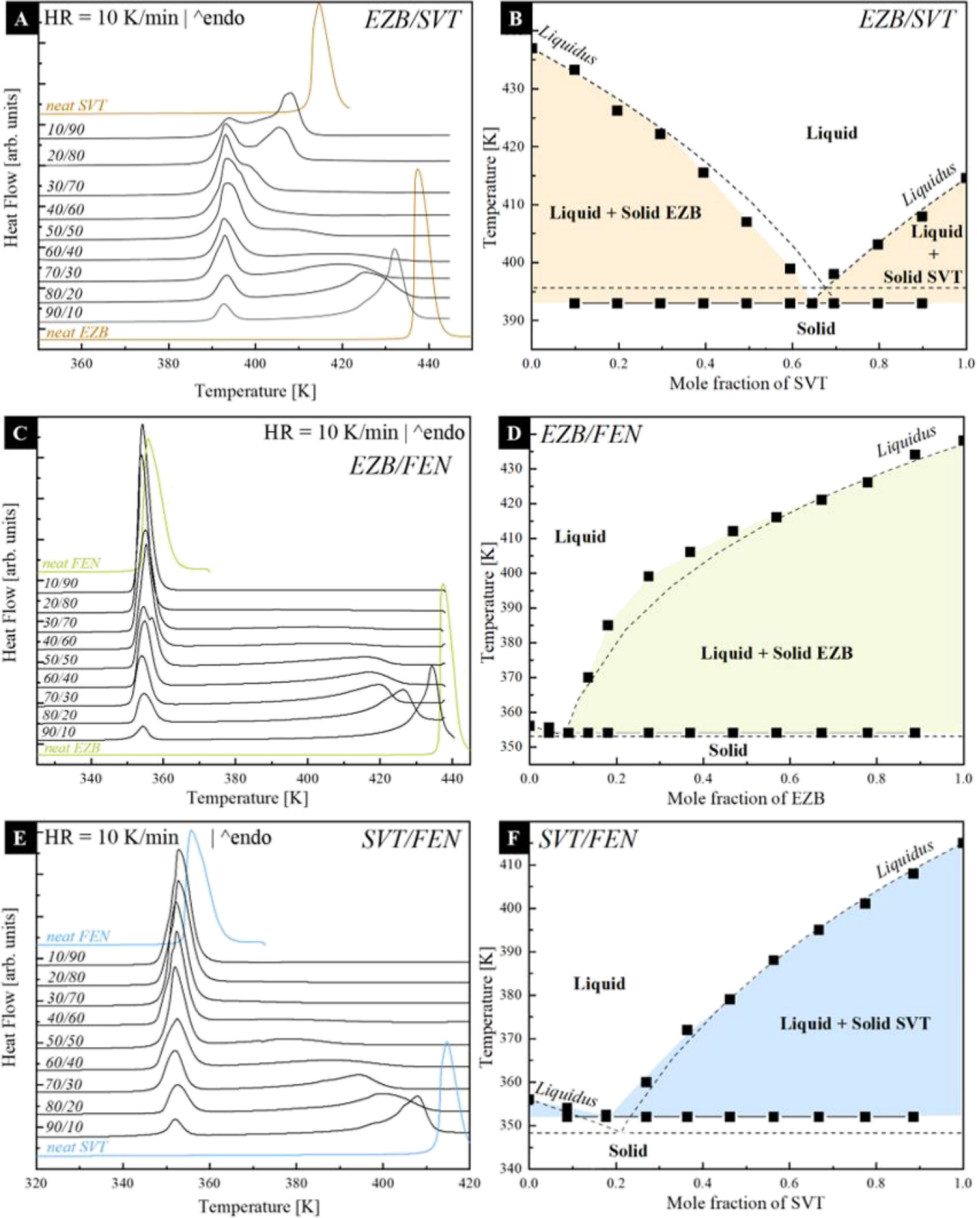
DSC thermograms of the crystalline physical mixtures of: (a) ezetimibe–simvastatin (EZB/SVT), (c) ezetimibe–fenofibrate (EZB/FEN), and (e) simvastatin–fenofibrate (SVT/FEN) together with their phase diagrams (b, d, f, respectively) which were constructed on the basis of both experimentally determined data (black squares) and theoretical data obtained based on the Schröder–Van Laar equation (black dashed lines).

To determine the eutectic concentrations of three binary systems: EZB/SVT, EZB/FEN, and SVT/FEN, the physical mixtures of these systems, with the mass ratio equal to 1:9, 2:8, 3:7, 4:6, 5:5, 4:6, 3:7, 2:8, and 9:1, were investigated utilizing differential scanning calorimetry (DSC). During these experiments, the samples were heated up from 303 to 445, 440, or 420 K in the case of EZB/SVT, EZB/FEN, and SVT/FEN, respectively. The obtained results together with the DSC curves of neat APIs are presented in panels a, c, and e of Figure 2. As can be seen, the thermograms are typical for eutectic systems. In most cases, one can observe the presence of two melting endotherms.^43^ The onset temperature of the first peak (i.e., the one located at a lower temperature) does not change with the mixture content. This temperature is called the solidus temperature below which the mixture is in its solid—fully crystalline—state. The temperature of the second melting endotherm shifts toward or away from the solidus temperature, depending on the drug concentration. Above this second peak—called liquidus temperature—the binary mixtures are in the fully molten state.^44^ By analyzing the recorded thermograms, the melting temperatures of the binary drug–drug mixtures were determined. The obtained results were then used to construct the phase diagrams (see black squares in Figure 2b,d,f).

As can be seen, all three investigated binary drug–drug compositions form eutectic systems. The concentration corresponding to a single melting endotherm is 35 wt % of EZB (i.e., *x*_EZB_ = 0.645), 10 wt % of EZB (i.e., *x*_EZB_ = 0.089), and 20 wt % (i.e., *x*_SVT_ = 0.177) of SVT for EZB/SVT, EZB/FEN, and SVT/FEN, respectively. In addition to the experimentally determined mole fraction dependence of liquidus temperatures presented in Figure 2b,d,f as black points, the theoretical values were calculated by means of the Schröder–Van Laar equation defined as follows:^44,45^
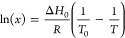
1where Δ*H*_0_ represents the heat of fusion (J·mol^–1^), *T*_0_ is the melting point of one of the pure drugs in the mixture, *T* is the melting point of the binary mixture at a specific mole fraction, *x*, and *R* represents the gas constant. The theoretically obtained liquidus curves are presented as dashed lines in panels b, d, and f of Figure 2. Both experimental and theoretical mole fraction dependences of liquidus temperature are in good agreement, while the error of the eutectic points determined from both these methods is less than 3.5%. Because the eutecticity of EZB/FEN and SVT/FEN systems is not so obvious as it is in the case of EZB/SVT more detailed data are shown in the Supporting Information (see Figures S1 and S2).

### 3.2 Determination of the Ternary Eutectic EZB–SVT–FEN System

**Figure 3:**
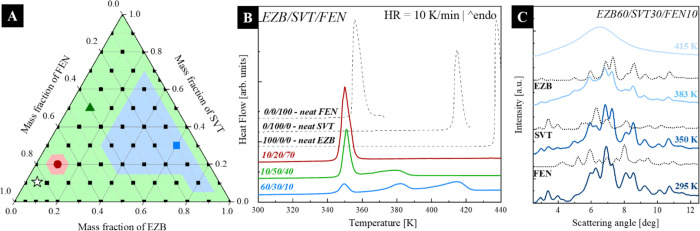
(a) Ternary plot illustrating by points the investigated compositions of EZB/SVT/FEN; colors represent the concentration areas where during heating one, two, or three melting endotherms were registered (one—red area, two—green area, three—blue area); (b) the DSC thermograms of the representative systems from those three areas (c) the XRD patterns of the EZB/SVT/FEN sample for 60/30/10 concentration as a function of temperature from 295 to 415 K. Diffractograms for neat crystalline FEN, SVT, and EZB are also shown. Melting of FEN, SVT, and EZB is observed as the Bragg peaks disappear corresponding to their crystalline phases at 350, 383, and 415 K, respectively.

In order to determine the ternary eutectic point of the EZB/SVT/FEN system, 37 mixtures having various concentrations of these drugs have been prepared and investigated utilizing DSC. Points in Figure 3a represents the investigated concentrations. During these experiments, samples were heated up from 298 to 440 K with a rate of 10 K/min. The selected DSC thermograms of the representative samples are presented in Figure 3b together with the thermograms of neat APIs. As can be seen, depending on the drug content, the thermogram of EZB/SVT/FEN might reveal one, two, or three endothermal events. The concentrations at which three thermal events were observed is marketed in panel a of Figure 3 as a blue area. The green area represents concentrations at which two melting endotherms were registered, while the red color corresponds to the eutectic concentration, revealing a single sharp thermal event. By performing a temperature-dependent XRD experiment on the sample for which the three thermal events were registered, it was possible to identify that the first thermal event corresponds to the melting of FEN, the second to the melting of SVT, while the third to the EZB’s melting (see panel c of Figure 3). Herein, it is worth emphasizing that the formation of co-crystals of the EZB/SVT/FEN system can be excluded based on the performed thermal analysis. It is because the cocrystals melt in the temperature range between the neat component melting.^46−48^ In comparison, all investigated thermograms reveal one melting endotherm having an onset at a lower temperature than the melting of each neat sample (compare the thermograms of neat APIs and the representative mixtures presented in panel b of Figure 3).

**Figure 4:**
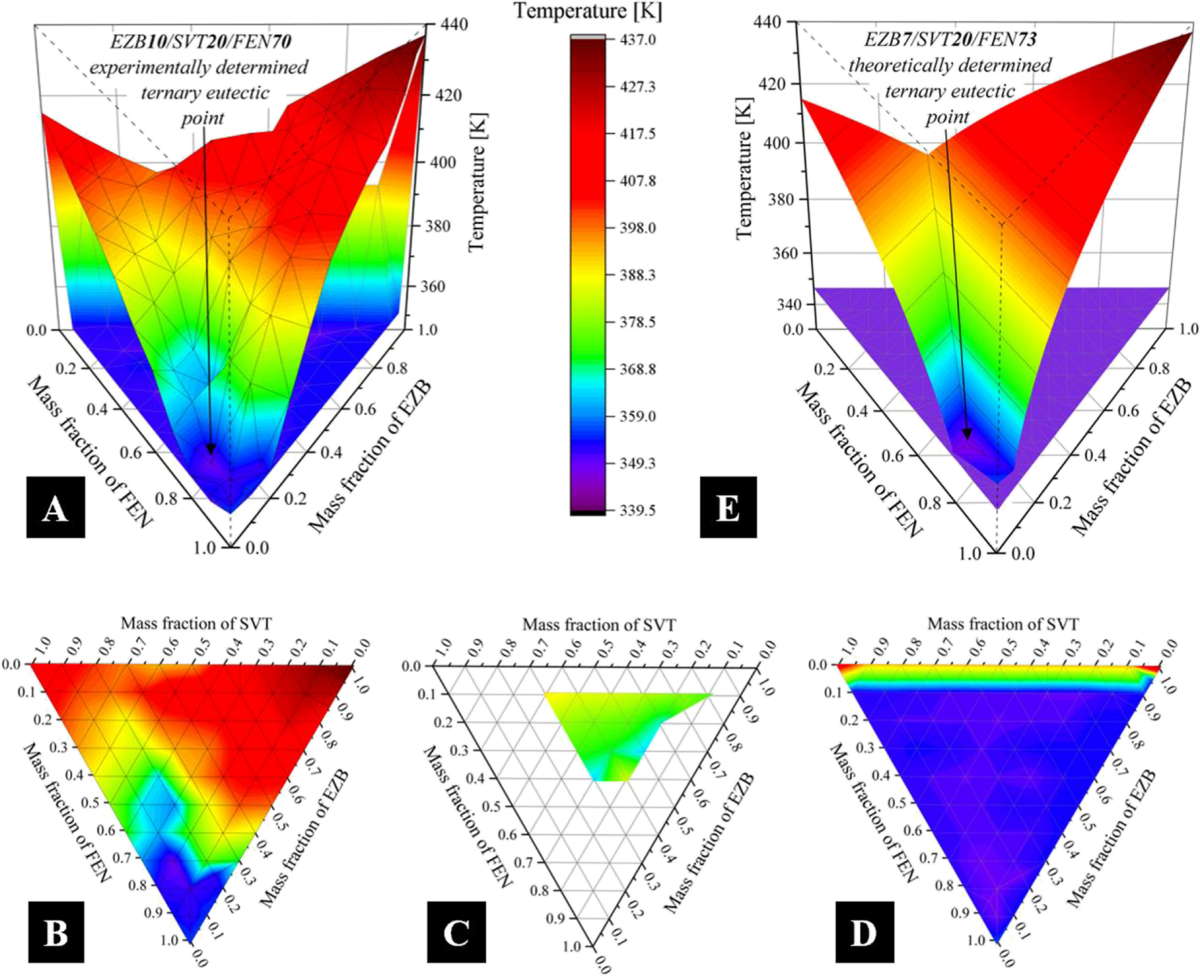
(a) 3D phase diagram of the EZB/SVT/FEN system constructed based on the experimental data; (b-d) 2D layers represents three melting areas presented in panel a; (e) 3D phase diagram of the EZB/SVT/FEN system constructed based on the theoretical data.

The 3D phase diagram constructed based on the experimental data is presented in panel a of Figure 4. To better visualize the concentration dependence of melting temperatures, the graph was divided into three 2D layers (see panels b, c, and d of Figure 4). From the ternary phase diagram, the eutectic point of EZB/SVT/FEN was found at a 10/20/70 mass ratio.

Since the precision in the determination of the eutectic point by the experimental method is associated with the number of investigated points (samples), the liquidus curves of the investigated system have also been calculated from the enthalpy of fusion and melting point of the neat components by the Schröder–Van Laar equation (see eq 1). The predicted concentration dependence of the liquidus temperature and, consequently, ternary eutectic point of the EZB/SVT/FEN system are presented as a 3D phase diagram in panel e of Figure 4. Comparing the ternary eutectic concentration determined from the experimental and theoretical approach, one can note that both these methods give similar results (i.e., there are no more than 5% differences between theoretically and experimentally determined eutectic concentration). The obtained results clearly indicate that during the combined therapy of EZB/SVT/FEN, which was proposed by Farner et al.,^2^ the eutectic compositions were not employed. Nevertheless, it is worth noting that the concentration of this system (i.e., EZB5.3/SVT10.5/FEN84.2) is close (±15%) to the concentration of the eutectic mixture determined in this paper. Consequently, it would be extremely interesting to check the therapeutic effectiveness of the EZB10/SVT20/FEN70 (eutectic) system for which the solubility, dissolution rate, and bioavailability should be better than the one proposed as a therapeutic. Nevertheless, because another competing method to the eutectic formation method to improve the solubility of BCS class II pharmaceuticals is amorphization, in the further part of the paper we will convert ternary EZB/SVT/FEN systems to the amorphous form and investigate its physicochemical properties.

### 3.3 Thermal Properties of Ternary Amorphous EZB/SVT/FEN Systems

**Figure 5:**
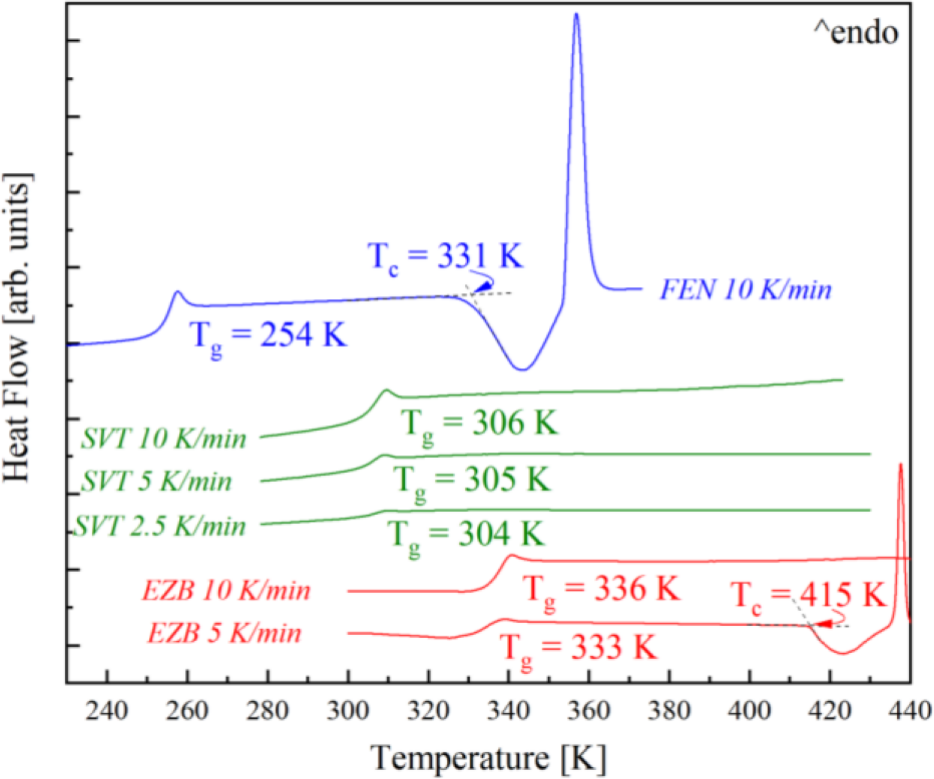
DSC thermograms of neat amorphous FEN (blue line), SVT (green lines), and EZB (red lines) measured with heating rates of 10, 5, and 2.5 K/min.

The quench-cooled neat amorphous EZB, SVT, and FEN, when measured by means of DSC with a rate of 10 K/min, have a glass transition (*T*_g_) at temperature equal to 336, 306, and 254 K, respectively (see Figure 5). Comparing the thermograms of the neat APIs, one can note that only FEN re-crystallizes during further heating with this rate, i.e., on the DSC curve both the exothermal event associated with recrystallization as well as the endothermal process connected with melting of the devitrified fraction of the samples are visible. To assess the tendency toward the recrystallization of the other APIs, slower heating rates, equal to 5 and 2.5 K/min, were employed. As can be seen the intermediate tendency toward recrystallization reveals EZB. Namely, the recrystallization of this API was registered at 415 K, when measured with a rate of 5 K/min, while, the greatest physical stability shows SVT, because no exothermal event was obtained even at 2.5 K/min heating rate.

**Figure 6:**
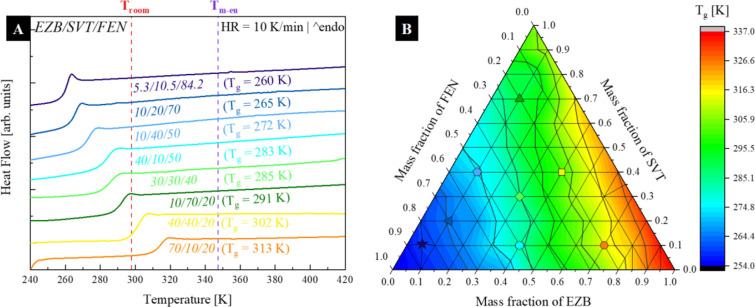
(a) DSC thermograms of eight representative ternary amorphous EZB/SVT/FEN systems, (b) the concentration dependence of the glass transition temperature of the EZB/SVT/FEN system.

To find out how the thermal properties of the mentioned pharmaceuticals vary when the drugs are mixed together, 37 ternary amorphous mixtures containing various mass ratios of EZB, SVT, and FEN were measured using the DSC technique. All the measurements were carried out from 240 to 440 K at the rate of 10 and 5 K/min. The DSC thermograms of eight of the most representative samples are depicted in Figure 6a. As can be seen, the mixtures containing EZB, SVT, and FEN are characterized by a single glass transition event suggesting a lack of phase separation. It has been many times reported that if components are not or only partially miscible, the DSC curve of its amorphous composition should reveal two or more separate *T*_g_ values (each belonging to the appropriate sample fraction).^49^ On the basis of all obtained data at the heating rate of 10 K/min, the concentration dependence of the glass transition temperature for the EZB/SVT/FEN system has been constructed, and is presented in Figure 6b. As can be seen, the *T*_g_ value strongly depends on the system concentration. It increases with increasing EZB or SVT content. If the mass fraction of FEN is higher than 0.3, the composition is characterized by *T*_g_ lower than the room temperature. On the other hand, the highest *T*_g_ values possess the systems having a mass fraction of EZB and SVT higher than 0.7 and 0.3, respectively.

It should be pointed out that none of the tested samples showed a tendency toward recrystallization both at 10 and 5 K/min heating rates. This result indicates that the physical stability of neat FEN and EZB was improved by the presence of the other APIs, regardless of whether the antiplasticization or plasticization effect was exerted.

In the further part of this paper, we will precisely investigate how the physical stability of the amorphous form of the investigated APIs changes depending on the mixture concentration. For that purpose, two kinds of experiments have been performed. The first approach considers the fact that the physical stability of amorphous APIs strongly depends on its molecular mobility. Consequently, all systems were examined under the same isochronal conditions (i.e., τ_α_ = const.). The second approach takes into account that all pharmaceutical products are finally stored under standard storage conditions (i.e., *T* = 298 K and *RH* = 40%). Thus, the investigated systems were stored for longer period of time at that particular, well defined conditions, and every once in a while, their stability was investigated by means of the XRD technique.

### 3.4 Molecular Dynamics of the Selected Supercooled Ternary System of EZB/SVT/FEN

**Figure 7:**
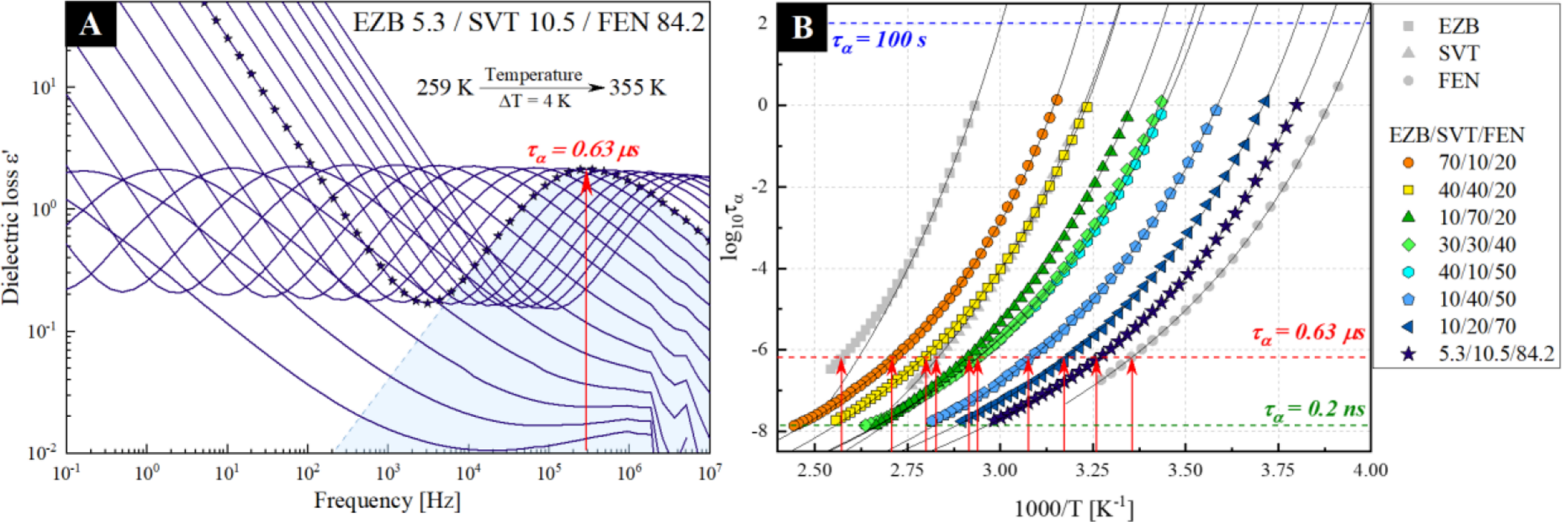
(a) Dielectric loss spectra collected above the glass transition temperature of ternary amorphous EZB 5.3/SVT 10.5/FEN 84.2 systems. (b) Temperature dependence of the structural relaxation times determined by using the BDS technique for pure amorphous EZB (gray squares), pure amorphous SVT (gray triangles), pure amorphous FEN (gray circles), and their ternary mixtures that contain various mass ratios of: EZB 70/SVT 10/FEN 20 (orange circles), EZB 40/SVT 40/FEN 20 (yellow squares), EZB 10/SVT 70/FEN 20 (dark green triangles), EZB 30/SVT 30/FEN 40 (light green diamonds), EZB 40/SVT 10/FEN 50 (cyan hexagons), EZB 10/SVT 40/FEN 50 (light blue pentagons), EZB 10/SVT 20/FEN 70 (dark blue triangles), and EZB 5.3/SVT 10.5/FEN 84.2 (navy stars). Solid black lines are the VFT_1_ fits, dashed red line marks the region, where τ_α_ = 0.63 μs, while red arrows represent the temperatures which corresponds to τ_α_ = 0.63 μs for each sample.

In this section, the molecular mobility of eight selected ternary systems of EZB/SVT/FEN was investigated. The selection considered the most representative concentrations (see points in Figure 6b) among which both the ternary eutectic and therapeutic systems were included. The aim of these experiments was twofold. On the one hand, the effect of additives on the molecular dynamics and the tendency toward recrystallization of the neat APIs were investigated. While on the other hand, the results of these studies allow us the proper selection of the storage conditions of the samples in the further stability tests in such a way that the systems are characterized by a constant relaxation time. To investigate the differences in the molecular mobility of the investigated ternary amorphous systems, the dielectric loss spectra of these samples were measured by means of broadband dielectric spectroscopy (BDS). The dielectric measurements were performed in the wide frequency (from 10^–1^ to 10^7^ Hz) and temperature range (from *T* = *T*_g_ to *T = T*(τ_α_ = 0.2 ns). The representative spectra of the ternary EZB/SVT/FEN composition having documented the excellent therapeutic effect on mixed hyperlipidemia treatment are shown in Figure 7a.

The dielectric loss spectra of all investigated ternary systems exhibit two features: the dc-conductivity associated with the translational motions of ions and the structural (α) relaxation process related to the cooperative rearrangement of the drug molecules. As shown in Figure 7a, the α-relaxation mode moves toward a higher frequency with increasing temperature, indicating increased molecular mobility. It is also worth noting that none of the investigated systems reveal a drastic drop in the intensity of the structural relaxation peak during the heating procedure. This indicates an absence of the cold crystallization of the mixtures, which is especially interesting since, according to the literature reports, neat amorphous EZB and FEN, during similar dielectric experiments, would recrystallize.^34,50,51^ The onset of the recrystallization process was registered at temperatures at which the maximum of the α-relaxation was located at a frequency from the range 10^4^–10^5^ Hz. Since devitrification of the APIs from the ternary mixtures was not observed, one can conclude that the enhancement of the physical stability is mainly driven by a thermodynamic factors (i.e., configurational entropy, enthalpy, or Gibbs free energy). If a kinetic factor (i.e., molecular mobility) would play here a key role, one could expect the presence of the recrystallization at similar, i.e., isochronal, conditions as in the case of neat APIs.

From the analysis of dielectric loss spectra, the temperature dependencies of the structural relaxation times (τ_α_(*T*)) for all investigated ternary compositions were obtained. The comparison of the τ_α_(*T*) dependencies of the measured ternary systems and neat APIs is presented in Figure 7b. The relaxation times were determined from fitting of dielectric spectra by the Havriliak–Negami (HN) function with the dc conductivity term, which is defined as follows:^52^
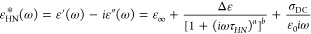
2where ε*_∞_* is the high-frequency limit permittivity, ε_0_ denotes the permittivity of vacuum, Δε is the dielectric strength, ω is equal to 2π*f*, τ*_HN_* is the HN relaxation time, and *a* and *b* represent symmetric and asymmetric broadening of the relaxation peak. By employing the determined fitting parameters, we finally calculated the *τ*_α_ values utilizing the following equation:
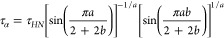
3

Usually, in the supercooled liquid region, the τ_α_(*T*) shows non-Arrhenius behavior and might be well parameterized by the Vogel–Fulcher–Tammann (VFT) equation:^53−55^
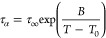
4where τ*_∞_*, *T*_0_, and *B* are the fitting parameters. Parameter τ_∞_ is a pre-exponential factor denoting the upper limit of temperature for *τ*_α_, which is correlated to the vibrational frequency (∼10^–11^ to 10^–14^ s). *T*_0_ is the Vogel temperature, which corresponds to the state with infinite relaxation time, and *B = DT*_0_, where *D* quantifies the deviation from the simple Arrhenius behavior. Since it has been previously noted that the temperature evolution of structural relaxation time of neat EZB does not conform over the entire experimental temperature range to a single VFT equation,^34^ thus prior to the fitting procedure each τ_α_(*T*) dependence was analyzed by the derivative method proposed by Stickel.^56^ This approach requires the employment of the operator [*d*(log(τ_α_))/*dT*]^−1/2^ = (*T* – *T*_0_)*B*^–1/2^, which linearized the VFT equation. Consequently, it was easy to determine the temperature regions in which the experimental data were well parameterized by a single VFT. This method confirmed that in almost all investigated ternary systems the obtained data had to be fitted using two VFT functions to cover the entire temperature range (except two concentrations with eutectic and therapeutic properties, i.e., EZB10/SVT20/FEN70 and EZB5.3/SVT10.5/FEN84.2). The temperature at which the VFT_1_ and VFT_2_ intersect (*T*_cross_), and all the fitting parameters are shown in Table 1. Additionally, the VFT_1_ fits are presented in Figure 7b as solid lines.

**Table 1:** Comparison of the Values of *T*_g_ Obtained from DSC and BDS, Fragility Parameters, VFT_1_ Fitting Parameters, *T*_cross_, and *T*(τ_α_ = 0.63 μs) for Neat Amorphous EZB, SVT, and FEN Drugs, and Their Ternary Amorphous Mixtures at Mass Ratios 70/10/20, 40/40/20, 10/70/20, 30/30/40, 40/10/50, 10/40/50,10/20/70, and 5.3/10.5/84.2

sample	*T*_g(DSC)_ [K]	*T*_g(BDS)_ [K]	*m*_p_	τ_∞_ [s]	*B*	*T*_0_ [K]	*T*_cross_ [K]	*T*(τ_α_ = 0.63 μs) [K]
EZB	336	333	93	–11.81 ± 0.03	1130 ± 11	301.1 ± 0.3	355	388
SVT	305	303	91	–15.68 ± 0.13	2386 ± 51	244.0 ± 0.9		353
FEN	254	251	95	–13.52 ± 0.17	1465 ± 10	209.3 ± 1.0		298
70/10/20	313	311	102	–12.51 ± 0.08	1483 ± 30	266.5 ± 0.7	395	369
40/40/20	302	301	104	–12.93 ± 0.12	1576 ± 41	256.4 ± 1.0	382	357
10/70/20	291	290	98	–13.78 ± 0.09	1769 ± 33	242.4 ± 0.7	368	343
30/30/40	285	284	97	–12.75 ± 0.10	1514 ± 36	240.1 ± 0.8	355	340
40/10/50	283	282	95	–12.79 ± 0.08	1564 ± 27	237.1 ± 0.6	361	340
10/40/50	272	271	98	–13.18 ± 0.14	1553 ± 46	277.7 ± 1.0	344	325
10/20/70	265	263	97	–12.57 ± 0.09	1346 ± 25	223.1 ± 0.6		315
5.3/10.5/84.2	260	257	99	–12.55 ± 0.04	1280 ± 13	219.0 ± 0.3		307

Because of the fact that VFT_1_ well describes the data in the region below the crossover temperature, it was utilized to estimate the kinetic glass transition temperature of each ternary system. The *T*_g_ value from BDS experiments was obtained using the commonly known definition *T*_g_ = *T*(τ_α_ = 100 s). These values are collected in Table 1, together with the *T*_g_ values determined from DSC. As can be noted, the glass transition temperature values determined by using two different methods slightly differ. The observed discrepancy between the kinetic and the calorimetric *T*_g_ values is associated with the different heating rates used in these experiments.

Finally, the τ_α_(*T*) dependences, determined from the dielectric data, were utilized for selecting the appropriate temperature conditions for further short-term physical stability studies performed at elevated temperature conditions. For that purpose, the straight line has been drawn at a fixed structural relaxation time equal to 0.63 μs (see the red dashed line in Figure 7b). Temperatures determined from the intersection of the drawn line with the temperature dependences of the samples structural relaxation times are collected in Table 1.

### 3.5 Physical Stability Studies of Selected Supercooled Ternary Systems Containing EZB, SVT, and FEN under Isochronal Temperature Conditions

**Figure 8:**
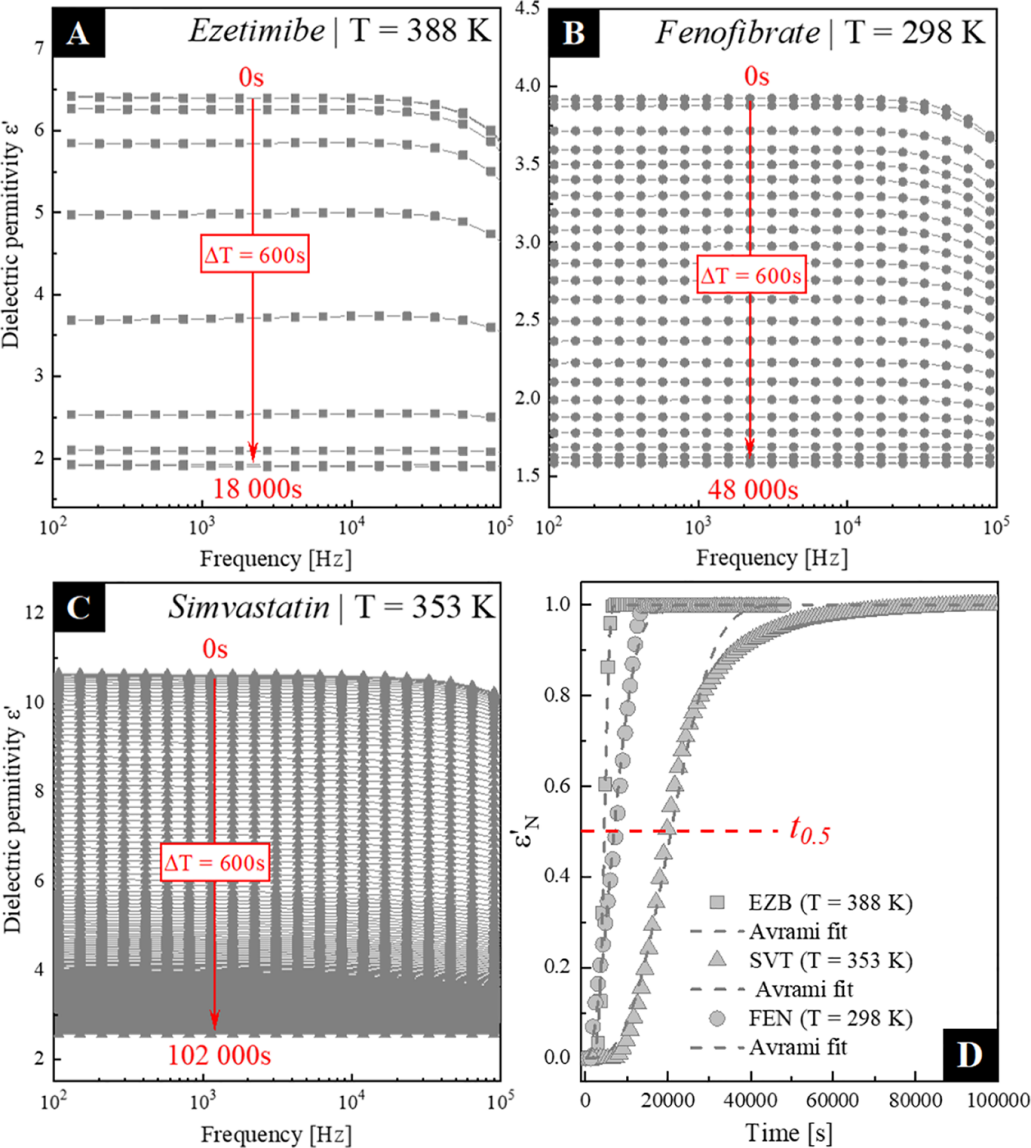
(a-c) Dielectric spectra of the real parts of the complex dielectric permittivity collected during the time-dependent isothermal experiment of: (a) EZB performed at 388 K, (b) FEN performed at 298 K, and (c) SVT performed at 353 K. (d) normalized dielectric constants ε′_N_ of EZB (squares), SVT (triangles), and FEN (circles) as a function of time from crystallization processes occurring at *T* = *T*(τ_α_ = 0.63 μs).

In this section, the physical stability of neat APIs and five ternary amorphous EZB/SVT/FEN systems were investigated and compared with each other. All samples were measured under temperature conditions at which their structural relaxation time (τ_α_) is equal to 0.63 μs. This particular condition was chosen based on previously published physical stability data of the neat EZB and FEN. According to the recalled data, EZB and FEN were stored at a temperature at which τ_α_ = 0.63 μs should fully recrystallize after ca. 2.5 and 4.5 h, respectively.^34,51^ First, to make sure that the physical stability of neat EZB and FEN is independent of the used batch as well as to investigate the recrystallization tendency of neat SVT stored at *T = T*(τ_α_ = 0.63 μs), the isothermal time-dependent dielectric experiments of these three neat APIs were performed. During these measurements, the spectra of the complex dielectric permittivity ε*(ω) = ε′(ω) – *i*ε″(ω) were investigated at specified time intervals of 600 s. By utilizing the dielectric spectroscopy, the recrystallization can be followed in both the real (ε*′*) and imaginary (ε*″*) parts of the complex dielectric permittivity, reflected by a decrease of the static permittivity (ε_s_) and reduction of the loss peak intensity with time, respectively.^57,58^ For our purpose, the real part of complex dielectric permittivity was selected for further analysis. The obtained results are shown in Figure 8a-c.

As can be clearly seen, EZB recrystallizes the fastest among the tested neat APIs, while SVT is characterized by the highest physical stability. The progress of crystallization is usually analyzed in terms of the normalized real permittivity (ε’_N_) defined as follows:^59−61^
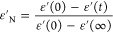
5where ε′(0) is the initial static dielectric permittivity, ε′(∞) is the long-time limiting value, and ε′(t) is the value at time *t*. The comparison of the normalized data plotted versus time is shown in panel d of Figure 8. The obtained results indicate that the crystallization half-life time (*t*_0.5_) of neat EZB, FEN, and SVT stored at a temperature at which τ_α_ = 0.63 μs is equal to 79, 114, and 330 min, while the entire devitrification takes 2, 4, and 25 h, respectively. Since the determined physical stability times of both EZB and FEN are in good agreement with the previously published data, one can conclude that the sample batch has no impact on the tendency toward recrystallization.

**Figure 9:**
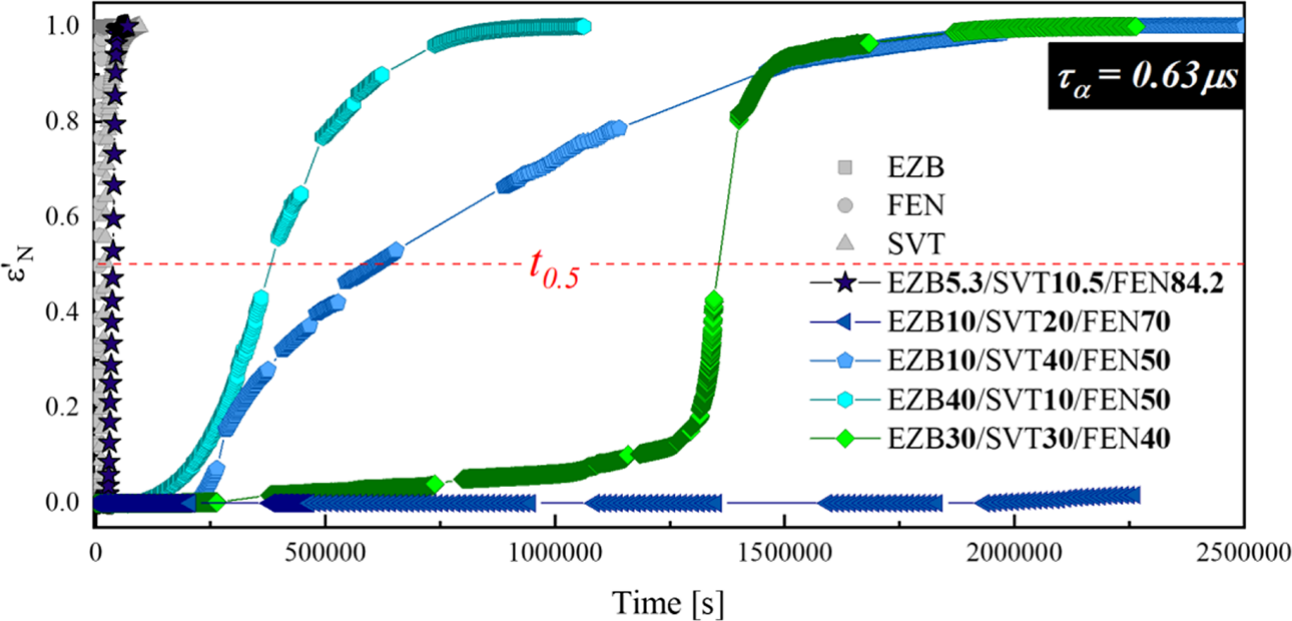
Normalized dielectric constants ε′_N_ of neat amorphous EZB (gray squares), SVT (gray triangles), and FEN (gray circles) as well as their ternary amorphous systems containing 5.3/10.5/84.2 (navy stars), 10/20/70 (dark blue triangles), 10/40/50 (light blue pentagons), 40/10/50 (cyan hexagons),and 30/30/40 (green diamonds) mass ratio of EZB/SVT/FEN as a function of crystallization time occurring at *T* = *T*(τ_α_ = 0.63 μs).

Knowing the physical stability of neat APIs at the chosen isochronal conditions, we performed similar physical stability experiments for the ternary compositions. The concentrations selected for these studies correspond to the eutectic concentration (EZB10/SVT20/FEN70), therapeutic concentration proposed by Farnier et al. (EZB5.3/SVT10.5/FEN84.2)^2^ and three representative concentrations which τ_α_(*T*) are presented in Figure 7b as cyan hexagons, light green diamonds, and light blue pentagons (EZB40/SVT10/FEN50, EZB10/SVT40/FEN50, and EZB30/SVT30/FEN40, respectively). The other three representative concentrations (EZB10/SVT70/FEN20, EZB40/SVT40/FEN20, and EZB70/SVT10/FEN20) were not tested since temperatures at which their τ_α_ = 0.63 μs are higher than the solidus temperature. In order to determine the temperature appropriate for each investigated sample (i.e., temperature at which τ_α_ = 0.63 μs) we used molecular dynamics data, described in the above section, presented in Figure 7b. Since the time of physical stability of the ternary system turned out to be much longer than for neat APIs, the samples were left for a longer period of time in the laboratory ovens and from time to time, they were connected to the dielectric spectrometer in order to register the progress of their recrystallization. When the devitrification process ceased, the obtained data were normalized in the same fashion as in the case of neat APIs. The kinetic curves obtained for all investigated samples, i.e., both neat APIs and their five ternary compositions, are compared in Figure 9.

As can be seen, the ternary composition having a therapeutic concentration of the drugs (EZB 5.3/SVT 10.5/FEN 84.2) recrystallizes the fastest among all investigated systems. The half-life time of this compositions is equal to 10.5 h, while the process ends after 13.5 h. The high content of FEN is probably the main reason for the quicker devitrification of this system in comparison to the neat SVT. It is worth noting that the other ternary compositions become fully crystalline after a significantly longer time than any investigated neat API. This result, without a doubt, confirms the positive effect of combining the tested pharmaceuticals on the physical stability of their amorphous form.

**Figure 10:**
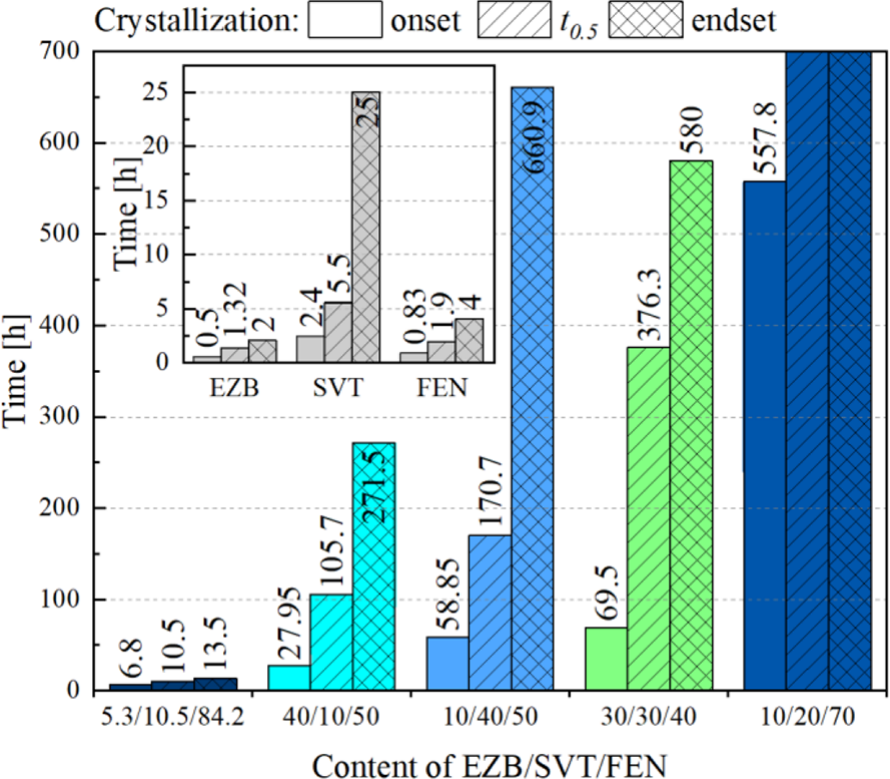
Comparison of the crystallization onset, half-life time, and endset of neat amorphous EZB, SVT, and FEN, as well as their ternary amorphous systems containing 5.3/10.5/84.2, 40/10/50, 10/40/50, 30/30/40, and 10/20/70 mass ratio of EZB/SVT/FEN.

To better visualize and consequently properly compare the tendency of the investigated compositions toward devitrification, the times of crystallization onset, half-life, and endset have been presented in Figure 10.

As can be seen, EZB40/SVT10/FEN50 is the second-fastest crystallizing system among those investigated. This result is not surprising—the system contains as much as 40% of EZB and 50% of FEN, which under similar conditions, crystallize much faster than SVT (see the inset of Figure 10). The composition of EZB10/SVT40/FEN50 reflects this dependence even better. Namely, because of the much greater amount of SVT than in the previous case, its devitrification takes a much longer period of time. It is worth highlighting that the full crystallinity of this system is reached even later than for EZB30/SVT30/FEN40, which begins to devitrify ca. 10 h later than EZB10/SVT40/FEN50. The latter composition has a smaller amount of SVT than EZB10/SVT40/FEN50, which can easily explain why its crystallization process is shorter. The only composition for which physical stability cannot be explained in a similar, as the above, way is EZB10/SVT20/FEN70. It is worth recalling that this particular concentration corresponds to the eutectic system. Recently Kissi et al. showed that in the case of binary amorphous drug–drug compositions of indomethacin–naproxen, nifedipine–paracetamol, and paracetamol–celecoxib, the highest physical stability was observed at ratios analogous to the eutectic concentrations of the respective crystalline mixtures.^62^ Taking this fact into account, one might conclude that regardless of the amount of the ingredients, the specific interactions, which on the one hand, are responsible for the formation of eutectic in crystalline mixtures, play a significant role in improving the physical stability of their amorphous counterparts.

### 3.6 Long-Term Physical Stability Studies of Selected Ternary Amorphous Systems Containing EZB, SVT, and FEN at Room Temperature

**Figure 11:**
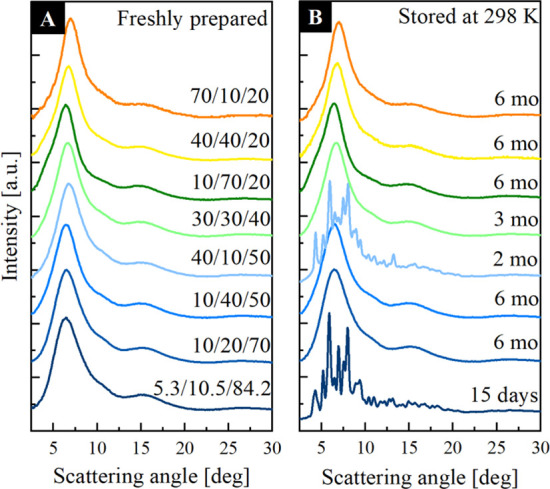
Representative XRD patterns of the selected ternary amorphous systems of EZB/SVT/FEN measured: (a) just after amorphization and (b) after long-term storage of the samples at *T* = 298 K.

In the previous section, the physical stability of the representative amorphous mixtures of EZB/SVT/FEN was studied at selected isochronal conditions (τ_α_ = 0.63 μs). Without a doubt, such experiments are essential from a cognitive point of view. Namely, they can help in finding a true molecular origin of the inhibition of recrystallization in the multicomponent amorphous systems. However, during such studies, each sample is stored at totally different temperature conditions (from 298 K in the case of neat FEN up to 388 K for neat EZB). Thus, from a practical point of view, it is also very important to investigate the physical stability of the ternary amorphous systems under conditions corresponding to the standard storage conditions (i.e., constant temperature *T*_room_ = 298 K and relative humidity *RH* = 40%). Having this in mind, we performed long-term physical stability studies at such conditions for eight selected ternary amorphous EZB/SVT/FEN systems (concentrations: 5.3/10.3/84.2, 10/20/70, 10/40/50, 40/10/50, 30/30/40, 10/70/20, 40/40/20, and 70/10/20). These measurements were realized by means of the XRD technique. The representative XRD patterns obtained during these experiments are presented in Figure 11. As can be seen in panel a of Figure 11, the diffractograms of all freshly prepared systems are characterized by broad, diffuse peak which confirms that the crystalline long-range order was destroyed in the investigated samples. This result proves that the tested ternary systems were fully amorphous just after the applied amorphization procedure. Next, the samples were left in a glow box, where humidity and temperature were monitored. The XRD diffraction patterns of these systems were collected once a week for the first month of the experiment, and then once a month for next 5 months. Data presented in panel b of Figure 11 indicate that the composition with a ratio of 5.3/10.3/84.2 (therapeutic system proposed by Farner et al.) begins to recrystallize the fastest among all investigated ternary systems. We noticed the first sign of its recrystallization just after 15 days after amorphization. It should be noted that destabilization of this system begins from FEN recrystallization.

The second sample for which the recrystallization was observed during the performed studies is 40/10/50 (EZB/SVT/FEN, respectively). The appearance of sharp Bragg’s peaks in the XRD diffraction pattern of this composition was registered 2 months after its amorphization. In this case, both recrystallization of FEN and EZB from the composition was noted. All other ternary amorphous systems do not reveal loss of their physical stability even after 6 months of storage. It is worth noting that the results obtained from long-term XRD studies, are relatively consistent with the stability studies performed at isochronal conditions by BDS. Namely, (i) the fastest recrystallization was observed for the EZB/SVT/FEN system with the concentration of 5.3/10.3/84.2, (ii) the second least stable system was the one with 40/10/50 concentration, and (iii) the ternary amorphous system having a concentration corresponding to the eutectic system (i.e., EZB10/SVT20/FEN70) is characterized by significantly higher physical stability than the concentrations described above. It should be pointed out that at the chosen experimental conditions (*T*_room_) some of the samples were measured in the supercooled liquid, while the others at their glassy state (compare the position of the red dashed line, which is marked *T*_room_ in Figure 6a with the distance from *T*_g_). Thus, from this type of study, it is hard to draw conclusions about the impact of the additives on the ternary amorphous system’s physical stability.

## 4 Conclusions

In this article, the phase diagrams of three binary EZB/SVT, EZB/FEN, and FEN/SVT systems, and one ternary EZB/SVT/FEN system were established by both experimental (calorimetric studies) and theoretical (Schröder–Van Laar equation) approaches. The obtained data reveal that all investigated systems can form eutectic concentrations. The concentration at which a single melting endotherm was registered in EZB/SVT, EZB/FEN, SVT/FEN, and EZB/SVT/FEN was 35 wt % of EZB, 10 wt % of EZB, 20 wt % of SVT, and 20 wt % of SVT and 70 wt % of FEN, respectively. It is worth highlighting that the measured ternary system’s eutectic point is in the vicinity (±15%) of the proposed therapeutic concentration of EZB/SVT/FEN. What can slightly explain why, in the proposed therapeutic concentration, the marvelous effect was noted in clinical trials. Based on the obtained data, it would be fascinating to check the medical effectiveness of the EZB10/SVT20/FEN70 (i.e., eutectic) composition.

The second aim of our studies was to convert the representative ternary EZB/SVT/FEN concentrations into amorphous form and investigate their physicochemical properties together with the impact of additives on the mixture’s physical stability. As has been demonstrated, all analyzed samples are characterized by a single glass transition event suggesting a lack of phase separation. *T*_g_ of the ternary systems changes from 254 to 333 K, depending on the component’s ratio. Since during the nonisothermal calorimetric studies, the lack of sample devitrification was observed, when both 5 and 10 K/min heating rate were employed, one can conclude that neat EZB and FEN’s physical stability improves in the presence of the other APIs. The time-dependent dielectric studies, which were performed when samples were stored at isochronal conditions, revealed that the amorphous system corresponding to the eutectic concentration is characterized by the highest physical stability. This result suggests that specific interactions, which on the one hand are responsible for the formation of eutectics in crystalline mixtures, play a significant role in improving the physical stability of their amorphous counterparts.

Because of the fact that pharmaceuticals are usually stored under standard storage conditions (i.e., *T*_room_ and RH = 40%), the last step of our studies was dedicated to the long-term physical stability studies of eight selected ternary compositions of EZB/SVT/FEN. The XRD experiment confirmed the previously observed high physical stability of the system EZB10/SVT20/FEN70 (i.e., having concentration corresponding to the eutectic mixture). The presented findings strongly suggest that it would be interesting to investigate the medical effectiveness of both ternary crystalline and ternary amorphous EZB/SVT/FEN systems having a eutectic concentration in comparison to one that is proposed in the literature. Furthermore, the obtained results open up a new way of selecting the therapeutic concentrations for combined therapies, a path that considers one additional variable: eutecticity.

## Supporting Information Available

DSC thermograms of the crystalline physical mixtures of simvastatin–fenofibrate (SVT/FEN); DSC thermograms of the crystalline physical mixtures of ezetimibe–fenofibrate (EZB/FEN) (PDF)
