# Supplementary material for: Ternary Eutectic Ezetimibe–Simvastatin–Fenofibrate System and the Physical Stability of Its Amorphous Form
Source: Mol Pharm. 2021 Aug 22;18(9):3588–600. doi: 10.1021/acs.molpharmaceut.1c00485 (PMC8424683; doi:10.1021/acs.molpharmaceut.1c00485)
Supplement: Supplementary file 1 — mp1c00485_si_001.pdf [file mp1c00485_si_001.pdf]

Ternary eutectic ezetimibe-simvastatin-fenofibrate system and the physical stability of its amorphous form

Justyna Knapik-Kowalczuk[1*], Daniel Kramarczyk[1], Karolina Jurkiewicz[1], Krzysztof Chmiel[2], Marian Paluch[1]

[1] *Faculty of Science and Technology, Institute of Physics, University of Silesia in Katowice, SMCEBI, 75 Pułku Piechoty 1a, 41-500 Chorzów, Poland;*

[2] *Department of Pharmacognosy and Phytochemistry, Medical University of Silesia in Katowice, School of Pharmacy with the Division of Laboratory Medicine in Sosnowiec, Jagiellonska 4, 41-200 Sosnowiec, Poland;*

**corresponding author: justyna.knapik-kowalczuk@us.edu.pl*

Supporting information

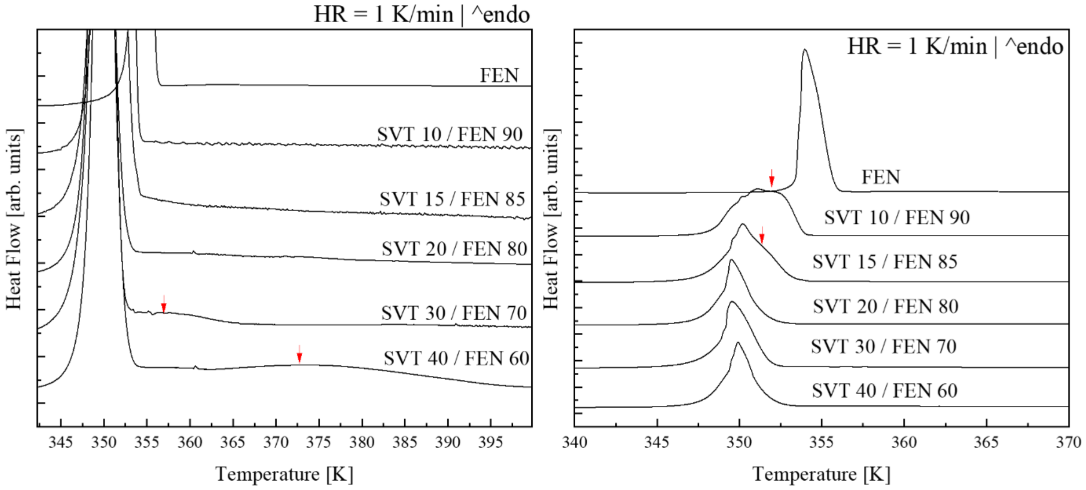

Figure S1. DSC thermograms of the crystalline physical mixtures of simvastatin-fenofibrate (SVT/FEN).

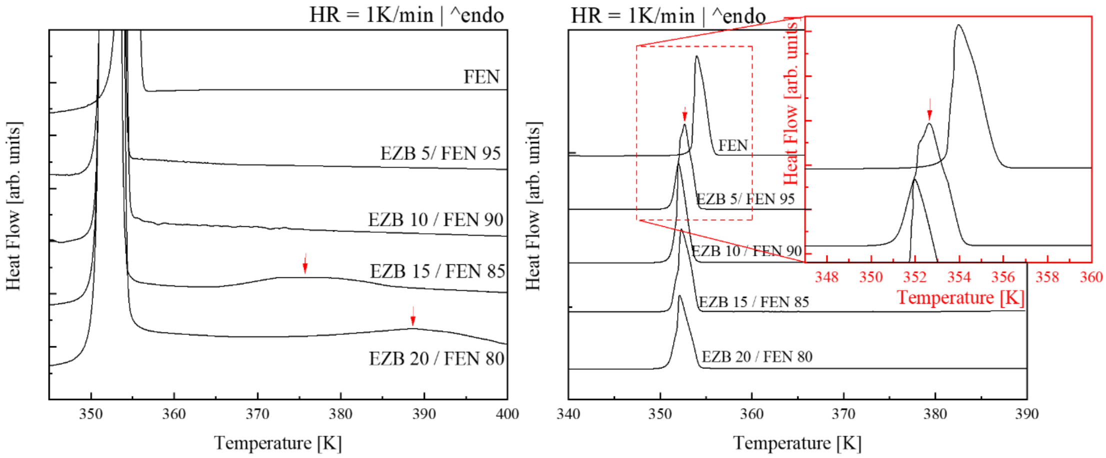

Figure S2. DSC thermograms of the crystalline physical mixtures of ezetimibe-fenofibrate (EZB/FEN).
